# Aspects of VLA-4 and LFA-1 regulation that may contribute to rolling and firm adhesion

**DOI:** 10.3389/fimmu.2012.00242

**Published:** 2012-08-02

**Authors:** Alexandre Chigaev, Larry A. Sklar

**Affiliations:** Department of Pathology and Cancer Center, University of New Mexico Health Sciences Center, AlbuquerqueNM, USA

**Keywords:** integrins, VLA-4, LFA-1, conformation, affinity, cell adhesion, rolling, tethering

## Abstract

Very Late Antigen-4 (CD49d/CD29, alpha4 beta1) and Lymphocyte Function-associated Antigen-1 (CD11a/CD18, alphaL beta2) integrins are representatives of a large family of adhesion receptors widely expressed on immune cells. They participate in cell recruitment to sites of inflammation, as well as multiple immune cell interactions. A unique feature of integrins is that integrin-dependent cell adhesion can be rapidly and reversibly modulated in response to cell signaling, because of a series of conformational changes within the molecule, which include changes in the affinity of the ligand binding pocket, molecular extension (unbending) and others. Here, we provide a concise comparative analysis of the conformational regulation of the two integrins with specific attention to the physiological differences between these molecules. We focus on recent data obtained using a novel technology, based on small fluorescent ligand-mimicking probes for the detection of integrin conformation in real-time on live cells at natural receptor abundance.

## Introduction

Integrins are a large family of adhesion receptors widely expressed on different cell types that participate in cell-matrix, or cell-cell interactions. These receptors can transmit signals in two directions. From the outside of a cell, ligation of the integrins results in the activation of a number of signaling pathways. Integrins can also serve as mechanosensors probing mechanical properties of the extracellular environment (Hogg et al., 2011). Ligation of other receptors, including different G-protein coupled receptors, cytokine, and chemokine receptors, Fc-receptors and others, can lead to the propagation of an inside-out signal toward the integrin (Hogg et al., 2011). This can result in a series of conformational changes within integrin molecules leading to a rapid increase or decrease of the integrin ligand binding affinity, molecular extension (unbending), movement of integrin domains (such as hybrid domain swing-out), and changes in integrin lateral mobility. These events directly modulate cell adhesion behavior (Askari et al., 2009).

In the peripheral blood, the majority of leukocytes exhibit a non-adhesive phenotype in which cells move freely with flowing blood. On these cells, integrins usually exist in a resting (inactive) non-adhesive state. On encountering soluble or immobilized ligands cellular behavior can be rapidly altered. Cells may roll on endothelial cells, arrest and firmly adhere, and transmigrate, leaving the blood vessel and crossing the endothelial barrier. Surprisingly, a number of steps in the cell adhesion cascade can be mediated by the same integrin molecule, existing in different conformational states that can be rapid and reversibly regulated through cellular signaling. Here we discuss recent insights into integrin conformational regulation. We will focus on two major leukocyte integrins (CD49d/CD29, Very Late Antigen-4, alpha4 beta1 integrin), and (CD11a/CD18, Lymphocyte Function-associated Antigen-1, alphaL beta2 integrin).

## Structural and functional differences between the two integrins

In the membranes of cells, integrins exist as heterodimers composed of one alpha and one beta subunit. In humans, 18 alpha and 8 beta subunits have been identified that combine to form at least 24 different heterodimers (Huhtala et al., 2005). An important feature of integrins is the presence of the so-called “inserted domain, or I-domain,” homologous to the von Willebrand factor A domain (vWFA). It can be found in every beta- (I-like domain), but only several alpha-subunits. This domain directly participates in the binding of the integrin ligands. Because of its homology to the vWFA alpha I-domains several groups prefer the term A-domain.

The alpha subunit with an inserted I-domain represents a late evolutionary acquisition. Even though teleost fish and several tunicata genomes contain integrin alpha subunits that have the inserted alpha I-domain, the leukocyte-specific integrin subunit orthologs, which include alpha D, alpha M, alpha X, and alpha L, are absent. Moreover, the beta2 integrin subunit that is known to form a dimer with each of these alpha subunits was also not found (Huhtala et al., 2005). Thus, it appears that the development of leukocytes, bearing diverse immune functions (as found in vertebrates), requires a set of leukocyte-specific integrin subunits. What would be the major physiological advantage to have these integrins? To answer this question we have to compare what is known about the physiological differences between alpha I-domain containing integrins (such as LFA-1) and leukocyte integrins lacking alpha I-domain, such as VLA-4.

According to the UniGene EST profile the overall expression patterns of integrin alpha 4 subunit (ITGA4) and integrin alpha L subunit (ITGAL) are very similar. These integrins are expressed in tissues associated with blood and lymphatic tissues. Blood, bone marrow, lymph, lymph nodes, spleen, and thymus are primary sites of expression. However, one major difference is that while LFA-1 expression is usually attributed to mature leukocytes, VLA-4 integrin is strongly expressed on CD34+ early hematopoietic stem progenitor cells (HSPCs). VLA-4 expression is critical for homing and retention of HSPCs, since blocking VLA-4-specific interactions using mAbs or small molecule antagonists is sufficient to induce cell mobilization into peripheral blood (Coulombel et al., 1997; Oostendorp and Dormer, 1997; Gazitt, 2004; Chigaev et al., 2011d). This observation is also confirmed by the fact that the expression of VLA-4 is more pronounced in “germ cell tumors.” (Compare the VLA-4 and LFA-1 UniGene EST profiles at http://www.ncbi.nlm.nih.gov/unigene/).

Two major integrin functions, related to cell adhesion, are usually assigned to VLA-4 and LFA-1. First, these integrins directly participate in cell arrest under flow, where firm adhesion is mediated by activated (high-affinity, unbent) integrins. Second, VLA-4 and LFA-1 contribute to cell-cell interactions that are critical for immune system responses. For example, both integrins play a part in the formation of immunological synapse, and participate in cell co-stimulation. There are also a number of differences between the two integrins related to both of these two functions. We postulate that these differences can be related to distinct structural and functional characteristics of these molecules, and thus, can provide a clue to the mystery of leukocyte-specific alpha I-domain-containing integrins.

Integrins are thought to be firm adhesion receptors. Historically, LFA-1 was one of the first integrins for which firm cell adhesion on activated cells was described. LFA-1 was unable to sustain cell rolling, and therefore, a selectin-mediated rolling step was envisioned to be necessary. However, under specific conditions that include LFA-1 mutagenesis, truncation, or treatment with allosteric antagonist rolling on LFA-1 can be observed (Table 1). This led to a multi-step recruitment paradigm, where cells will first roll on selectins, and after encountering activating stimuli they will arrest, and transmigrate (von Andrian et al., 1991; Springer, 1994). The discovery that in addition to firm adhesion VLA-4 can mediate cell tethering and rolling (Alon et al., 1995) represents the first indication of a functional difference between VLA-4 and LFA-1. More detailed analysis revealed that VLA-4 supports a number of adhesive interactions that are directly related but not limited to the maintenance of immune cells through hematopoiesis (Imai et al., 2010), as well as intrinsic immune responses. Thus, VLA-4 participates in chemokine-dependent cell arrest on endothelium, NK, and MKT cell recruitment to bone marrow, cell recruitment in response to bacterial infections, bacterial killing, etc. In contrast, LFA-1-dependent cell adhesion is critical for modulating adaptive immune responses that include T-B-cell interaction, Antigen Presenting Cell (APC)-T-cell interaction, regulation of TCR signaling, host-versus-graft reaction, binding, etc. (Table 1).

**Table 1:** **Examples of different functional roles of LFA-1 and VLA-4-dependent adhesive interaction**.

**Function**	**LFA-1/ICAM-1 interaction**	**VLA-4/VCAM-1 interaction**
Tethering or rolling under shear flow	No tethering or rolling under shear flow; requires selectin-mediated rolling (Lawrence and Springer, 1991; von Andrian et al., 1991). No contribution to lymphocyte rolling on high endothelial venules (Warnock et al., 1998). Rolling can be artificially induced when mutated I domains or isolated wild type I domains are used (Salas et al., 2002). Small molecule allosteric antagonist XVA143 stimulates rolling on ICAM-1 (Salas et al., 2004).	Shown to mediate tethering or rolling (Alon et al., 1995; Berlin et al., 1995). Integrin activation is not required for tethering or rolling (Alon et al., 1995).
Engraftment of non-obese/severe combined immunodeficiency mice by human stem cells	Treatment with anti-LFA-1 antibodies caused partial inhibition of engraftment (by ~20%) (Peled et al., 2000).	Treatment with anti-VLA-4 antibodies prevented engraftment (Peled et al., 2000).
NK cell and NKT cell recruitment to bone marrow	LFA-1 does not participate (Franitza et al., 2004).	VLA-4 is critical for recruitment (Franitza et al., 2004).
Recruitment of cells to lungs during *Streptococus pneumoniae* infection	No LFA-1 contribution found (Kadioglu et al., 2011).	T cell recruitment solely dependent on VLA-4; neutrophil recruitment depends also on Mac-1 (Kadioglu et al., 2011).
Phagosome maturation in macrophages	Search for “LFA-1 AND phagosome maturation” in PubMed database returned no items.	VLA-4 (and VLA-5) are critical for phagosome maturation. Integrin-deficient macrophages have impaired bactericidal activity (Wang et al., 2008).
T-B-cell interactions *in vivo*	LFA-1/ICAM-1 interactions are critical for polyclonal B-cell activation in host-versus-graft model (Lopez-Hoyos et al., 1999).	Blocking of VLA-4 had no effect (Lopez-Hoyos et al., 1999).
Immunological synapse	LFA-1-dependent interaction represent an important part of immunological synapse, playing a role in adaptive immune responses (Dustin, 2008; Springer and Dustin, 2012).	Only a few papers describe involvement of VLA-4 in immunological synapse formation and signaling (Burkhardt, 2008; Carrasco and Batista, 2006).

Publications where similar integrin functions were reported are not included as the authors intentionally focused on functional differences between the two integrins.

Thus, another functional difference between the two integrins is related to their role in the immune system. It appears that VLA-4, representing an ancient integrin family expressed on leukocytes, is predominantly related to certain types of innate antigen-independent non-specific immune responses, where no significant role for LFA-1 is shown. LFA-1 is predominantly related to the signaling pathways, where antigen-dependent adaptive immunity plays a critical role (Table 1).

Furthermore, the appearance of leukocyte-specific alpha I-domain-containing integrins during evolution coincides with the emergence of the BCR-TCR-MHC-based adaptive immune system. The whole genome duplication that occurred at the dawn of jawed vertebrate evolution provides a mechanism for the emergence of novel genes that included a set of leukocyte-specific alpha and beta subunits. The “big bang” that created vertebrate adaptive immune system could be responsible for leukocyte integrin evolution as well (Flajnik and Kasahara, 2010). Thus, it is not surprising that leukocyte-specific alpha I-domain-containing integrins, such as LFA-1, are functionally linked to the adaptive immune system and BCR/TCR/MHC-related signaling pathways.

## Fluorescent probes as tools for integrin studies

We postulated that the physiological difference in the integrin function could be directly related to a primary integrin function: binding of the integrin ligand under different signaling conditions. To study the real-time regulation of integrin affinity and conformation, we developed a set of small fluorescent probes. [For VLA-4 see (Chigaev et al., 2001, 2003b, 2004) and for LFA-1 see (Chigaev et al., 2011b)]. These molecules were designed using small molecule integrin antagonists, developed by pharmaceutical companies, and have been shown to bind to the natural ligand binding sites. Therefore, these molecules mimic the binding of a natural ligand (Chigaev et al., 2003b; Zwartz et al., 2004), and because of an intrinsically higher affinity and commercial availability, these probes can be used in homogeneous assays to study rapid integrin conformational changes on live cell and in real-time (Chigaev et al., 2003a,b; Chigaev and Sklar, 2012). For example, for the detection of a real-time affinity change, the experimental concentration of the probe is required to be below the dissociation constant (*K*_*d*_) for its binding to the resting integrin, and above the *K*_*d*_ for the physiologically activated integrin. Therefore, the transition from the low affinity to the high affinity state after “inside-out” activation through a G-protein coupled receptor, leads to an increase in the binding of the probe that can be detected using a conventional flow cytometer. Physiological signaling pathways involving cAMP and cGMP that lead to integrin deactivation result in rapid probe dissociation (Chigaev et al., 2001, 2008, 2011a,b). Moreover, different integrin affinities can be detected through analysis of ligand dissociation rates. Slower rates correspond to states of higher affinity (Chigaev et al., 2003b).

Vertical extension of the VLA-4 integrin molecule can be detected using a FRET-based approach, where a fluorescent probe bound to the integrin headgroup serves as the donor, and octadecyl rhodamine B incorporated into the cell membrane, serves as the acceptor (Chigaev et al., 2003a,^2007^, ^2008^). Using these and other approaches (Chigaev et al., 2009) which depend upon the ability of the flow cytometer to discriminate fluorescent signals from a cell and the volume around it, a complex picture of conformational regulation of integrin has emerged.

## Integrin conformation in the regulation of integrin dependent cell adhesion

Integrins can exist in multiple conformational states. For LFA-1, at least three states that differ in ligand binding affinity (low, intermediate, and high affinity) have been reported. Moreover, application of an external force can lead to the stabilization of ligand binding [or “catch bond” (Kong et al., 2009)], while lateral shear force can significantly modify the adhesive properties of LFA-1 (Hogg et al., 2011). For VLA-4, the discovery of several distinct signaling mechanisms that can independently regulate the affinity of the ligand-binding pocket and molecular unbending (or extension, detected using FRET-based approaches), was an early indication of the conformational complexity of this non I-domain-containing integrin (Chigaev et al., 2007). Next, it was found that after inside-out activation through wild type Gαi-coupled GPCRs, ligand binding affinity and molecular extension exhibited distinctly different time courses (Chigaev et al., 2007). In contrast, the fact that VLA-4 deactivation through Gαs-coupled GPCRs only affected the affinity of the integrin ligand binding pocket (Chigaev et al., 2008), provided a plausible explanation for the differences in cell adhesion at rest and after cAMP-dependent integrin deactivation [see Figure 7A in Chigaev et al. (2008)]. The use of conformationally sensitive antibodies in real-time on living cells allowed us to answer several questions regarding the role of the hybrid domain movement during inside-out activation and ligand engagement as described below (Chigaev et al., 2009; Njus et al., 2009). Using this information we can reconstruct a model of integrin conformational states for a non I-domain containing integrin (VLA-4).

## Hybrid domain

On resting cells, in the absence of ligand, VLA-4 exhibits a low affinity, bent conformation with a hidden hybrid domain epitope (based on the lack of HUTS mAb binding). Although, the approach for assessing VLA-4 extension is based on FRET between the fluorescent ligand bound to the integrin headgroup and a membrane bound fluorescent acceptor, the observation that inside-out activation rapidly induces FRET signal unquenching suggests that at rest the VLA-4 headgroup is closer to the membrane. The inside-out activation through a Gα i-coupled GPCR in the absence of a ligand has only a small effect on hybrid domain movement. In this case, the short-term exposure of the HUTS epitope was maximal during the first 30 s after GPCR signal, and it was undetectable 4 min after activation based on the rate of HUTS Mab binding (Chigaev et al., 2009). Under similar conditions, the high affinity state of the VLA-4 ligand binding pocket was sustained for more than 15 min, in the presence of a non-desensitizing mutant of the GPCR (Prossnitz, 1997; Chigaev et al., 2007, 2011a). Thus, at least for VLA-4, no direct connection between exposure of the hybrid domain epitope [and an outward swing of a beta-1 subunit hybrid domain (Mould et al., 2003; Mould and Humphries, 2004)] with the high affinity activated state can be established.

Multiple VLA-4-specific compounds, with binding affinities spanning more than three orders of magnitude, were all capable of inducing exposure of the hybrid domain epitope (Njus et al., 2009). Moreover, a quantitative analysis of the fractional occupancy of the VLA-4 ligand binding pocket revealed that EC_50_s for the ligand-induced epitope exposure were virtually identical to the *K*_*i*_*s* determined in the competition assay with the fluorescent VLA-4 specific ligand. This suggests that occupancy of the VLA-4 ligand binding pocket by a direct (competitive) VLA-4 ligand is directly translated into HUTS epitope exposure, which can be also detected in real-time (Chigaev et al., 2009; Njus et al., 2009). This approach was recently adapted for the discovery of VLA-4 allosteric antagonists (see below) (Chigaev et al., 2011c,d).

## Extension and affinity

The inside-out activation through Gαi-coupled GPCRs induced rapid VLA-4 extension that can be detected using a FRET-based approach. In the presence of the ligand this created an extended conformation with an exposed hybrid domain epitope. However, in the case of wild type GPCRs the high affinity state existed only for a few minutes. After that, the binding affinity rapidly returned to a resting low affinity state. On the contrary, VLA-4 molecular extension detected using FRET persisted for several tens of minutes (Chigaev et al., 2007). This created a sustained low affinity extended state that was similar to the state induced by Gα s-GPCR induced deactivation (Chigaev et al., 2008) or the nitric oxide (NO) and cGMP-dependent signaling (Chigaev et al., 2011a). We envision that this state could sustain cell rolling. Significant similarity between cAMP and cGMP-dependent signaling mechanisms, together with a specific role of cAMP-dependent guanine-nucleotide-exchange factors for small GTPases (Rap1 and Rap2), which are implicated in integrin-mediated cell adhesion (Bos, 2006), suggest that cyclic nucleotides may represent a universal, and previously underestimated mechanism of integrin regulation.

Another VLA-4 state was observed after cell treatment with phorbol ester. Phorbol 12-myristate 13-acetate rapidly elevated VLA-4 affinity in a dose dependent manner. However, it failed to stimulate any extension of the molecule as detected using FRET. Moreover, the addition of calcium ionophore fully restored VLA-4 extension (Chigaev et al., 2007). This led us to hypothesize that cytoplasmic Ca^2+^ elevation is obligatory for molecular unbending, in contrast to the diacylglycerol-dependent step, which regulates the affinity of the ligand binding pocket. The recent finding that two adaptor proteins (talin-1 and kindlin-3) can independently regulate LFA-1 integrin extension and ligand binding affinity (Lefort et al., 2012) provides additional support for our current model (Chigaev et al., 2007).

## Integrin conformation and cell adhesion

How are multiple VLA-4 conformations translated into cell adhesive properties? To address this question, see the data summarized in Table 2. Two different approaches to study cellular behavior for differing VLA-4 conformations were used: (1) real-time cellular aggregation in solution in a VLA-4/VCAM-1 dependent cell adhesion model system (Zwartz et al., 2004), and (2) cell rolling in a parallel plate on low density recombinant human VCAM-1 (DiVietro et al., 2007).

**Table 2:** **Behavior of VLA-4 conformational states using several approaches**.

**Cell treatment, activation (pathway)**	**Small fluorescent ligand binding (LDV-FITC)^a^**			**Extension of the molecule (FRET-based assay)^b^**	**Real-time cell aggregation in solution^c^**		**Rolling on low density rhVCAM-1^d^**	
	**Association rate, *k*_on_**	**Dissociation rate, *k*_off_**	**Overall affinity**		**Initial rate of cell aggregation**	**Number of aggregates at steady state**	**Capture frequency**	**Tether duration**
Resting state	Fast	Fast	Low	Bent	Slow	Low	Low	Short
fMLFF, FPR activation (Gα i signal)	Fast	Slow	High	Extended	Rapid	High	High	Long
Phorbol ester (PKC)	Fast	Slow	High	Bent	Slow	High	Low	Long
fMLFF + Forskolin (Gα i and Gα s)	Fast	Fast	Low	Extended	Rapid	Medium	Low	Short

a Based on data from Chigaev et al. (2001, 2003b, 2007, 2008).

b Based on data from Chigaev et al. (2003a, 2007, 2008).

c Based upon Chigaev et al. (2007, 2008).

d Unpublished data.

The real-time analysis of cell aggregation in solution showed a strong correlation between the initial rate of aggregation and the extension of VLA-4 detected using FRET. On the other hand, the overall number of aggregates at steady state was related to the overall ligand-binding affinity that was largely determined by the dissociation rate of soluble ligand (LDV-FITC, *k*_off_, Table 2). It is worth noting that such an unambiguous result was possible since under the chosen experimental conditions only a small number of VLA-4/VCAM-1 bonds were needed to form and sustain cellular aggregates. According to an experimental estimate, in most cases this number was less than three bonds per aggregate (Zwartz et al., 2004). Thus, molecular extension seemed to facilitate the initial VLA-4-VCAM-1 ligand interaction and therefore, promote initial receptor engagement. Slow ligand dissociation, in the high affinity state stabilizes cellular interactions, and therefore, results in a larger number of cell aggregates.

In the parallel plate rolling assay at very low density of immobilized ligands, the formation of multiple consecutive “bonds” between receptor and its counter-structure is relatively unlikely. Therefore, under these experimental conditions the kinetics of transient tether formation and its dissociation provides insight into the functional consequences of nascent adhesive contacts (Grabovsky et al., 2000). We hypothesized that integrin extension, because of the exposure of the ligand binding site, directly affects the efficiency of tether formation. On the other hand, the affinity of the binding pocket determines the life-time of the integrin-ligand interaction and thus, regulates the duration of the adhesive event. To test this idea we studied tether frequency and duration for the four affinity states described previously (Table 2). The high affinity extended state of VLA-4 induced by stimulation through a Gαi-coupled receptor produced rapid accumulation of cells and long tether duration, when compared to the low affinity bent resting state. Phorbol ester treated cells showed low cell recruitment and long tether duration. This state was previously described as a high affinity bent conformation of VLA-4. Treatment with fMLFF/forskolin (intended to reproduce cAMP elevation through Gα s signaling), which generates a low affinity unbent (extended) state, showed tether duration similar to the resting cells. However, the tether frequency was unexpectedly low for an unbent conformation. This unanticipated result might result from our inability to accurately estimate the number of very short tethers and merits further investigation. Thus, the parallel plate data were generally consistent with the predicted behavior except for the low affinity extended state to promote efficient cell recruitment and adhesion (Chigaev et al., 2007).

**Figure 1:**
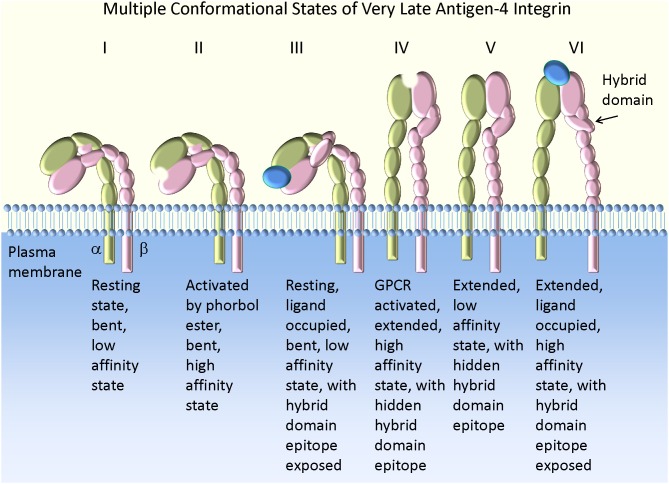
**Model of VLA-4 integrin conformation and affinity.** The bent low affinity state is observed on resting cells (I). Activation by phorbol ester creates a high affinity state lacking molecular extension as detected by a FRET-based approach (II). This conformation results in the slow accumulation of cell aggregates in suspension (Chigaev et al., 2007), with a low tether capture frequency but a long tether duration in the rolling assay (Table 2). The addition of VLA-4 specific ligand to resting cells leads to exposure of a hybrid domain (LIBS) epitope (III). However, the ligand binding affinity remains low [see Figures 2, 4 in Chigaev et al. (2009)]. This state is bent (or at least not fully extended) because a further molecular extension can be detected with a FRET-based approach (Chigaev et al., 2003a, 2004, 2007). Activation through a wild type Gα i-coupled GPCR induces a high affinity extended conformation (IV). This conformation results in the rapid accumulation of cell aggregates in suspension (Chigaev et al., 2007), with high tether capture frequency and long tether duration (Table 2). The low affinity extended (or at least partially extended) conformation (V) can be detected for several minutes after signaling from wild type Gα i-coupled GPCR, because of relatively faster desensitization of the ligand binding affinity than relaxation of the conformation (Chigaev et al., 2007). Conformation V may also result from consecutive stimulation through Gα i-coupled and Gα s-coupled receptors (Chigaev et al., 2008). In suspension this translates into rapid cell aggregation that reaches a steady-state intermediate between resting (I) and Gα i-coupled GPCR activated states (IV) [see Figure 7A in Chigaev et al. (2008)]. A low tether capture frequency and short tether duration was detected in the rolling assay (Table 2) (see text for details). The ligand occupied and extended high affinity state (VI) was detected after Gα i-coupled GPCR activation in the presence of ligand. The molecule affinity and extension were preserved by the use of a non-desensitizing GPCR mutant (Prossnitz, 1997; Chigaev et al., 2003a, 2007). This conformation results in the rapid formation of a large number of aggregates in cell suspension (Chigaev et al., 2008), with high tether capture frequency and long tether duration in the rolling assay (Table 2). The exposure of the hybrid domain (LIBS) epitope can be also used to determine VLA-4 ligand binding affinity for unlabeled ligands (Chigaev et al., 2009; Njus et al., 2009).

Taken together, the overall scheme of the VLA-4 conformation regulation can be generalized as follows (Figure 1). At rest, the low affinity bent conformation prevents cell tethering and rolling because of the positioning of the ligand binding site. If ligand engagement occurs, it would have a very short life time. However, it is also possible that a series of engagements of integrins or other receptors [selectins for example (Kuwano et al., 2010)] could provide a signal resulting in molecular extension. This could lead to rolling on an extended low affinity integrin. Rapid activation by Gα i-coupled GPCR induces a short-lived high affinity extended state (seconds to minutes), followed by a sustained extended low affinity state. If during the short period that VLA-4 engages its counter-structure, a long-lived tether will form. Under shear and external force this interaction can potentially be sustained for a longer period of time because of mechanical (catch bond) or signaling/cytoskeletal events. If no engagement of the integrin occurred, a low affinity extended state could be ideally suited for rolling under shear.

## LFA-1 conformation

A similar approach employing a small fluorescent ligand mimicking probe was used to study LFA-1 conformational regulation. Because several small molecules, direct and allosteric antagonists, are known to specifically bind to LFA-1, multiple fluorescent probes based, for example, on BIRT0377 (Larson et al., 2005), Genentech compounds (Gadek et al., 2002; Chigaev et al., 2011b), and others can be used.

Employing this approach, recent studies of LFA-1 conformational regulation revealed notable similarities and differences in the regulation of VLA-4 and LFA-1. In a manner analogous to VLA-4, LFA-1 can be rapidly activated by Gα i-coupled GPCRs, with the overall activation time-frame dependent on the rate of GPCR desensitization. Similar to VLA-4, LFA-1 can be rapidly deactivated by Gα s-coupled GPCRs. Also similar to VLA-4, modulation of the ligand dissociation rate can be observed for different LFA-1 affinity states (Chigaev et al., 2011b). However, unlike VLA-4, without inside-out activation (at rest), the binding of the fluorescent ligand to LFA-1 was extremely slow, at least 10 times slower than was expected for diffusion-limited binding. This suggests that an additional structural mechanism prevents rapid binding of the ligand to resting LFA-1. In the case of VLA-4 the binding of the ligand is unobstructed, and ligand binding rates are close to the rates expected for the diffusion-limited binding regardless of activation state, the *k*_on_ ranged from ~3–5 × 10^6^ M^−1^s^−1^ (Chigaev et al., 2001). We postulate that such a blocking mechanism explains the inability of native LFA-1 to support cell rolling, where the absence of its rapid engagement by the ligand in the inactive state leads to the requirement for the selectin-mediated rolling step (Table 1) (Chigaev et al., 2011b). A recent funding that rolling on E- or P-selectin induces the extended but not high-affinity conformation of LFA-1 through a signaling mechanism triggered by PSGL-1 engagement adds more complexity to the overall scheme of LFA-1 conformational regulation (Kuwano et al., 2010).

## Ligand binding rates, I-domain, and integrin physiology. is there a relationship?

For adhesion receptors, the kinetics of ligand-receptor interaction, which includes “bond” formation and its dissociation, is a critical factor that determines the type of adhesive interaction. The rapid forward rate (on-rate) is specifically important for cell rolling, because of the requirement for rapid molecule engagement under flow (Lawrence and Springer, 1991). As discussed above, the major difference between the two integrins is that the on-rates for the binding of small ligands to VLA-4 and LFA-1 are dramatically different. The on-rate for ligand binding to VLA-4 approaches the diffusion-limited binding rate for a ligand of similar size (Chigaev et al., 2003b). For the LFA-1-specific ligand, this rate is at least an order of magnitude slower. We believe that this LFA-1-specific kinetic property is directly translated into the well documented inability of LFA-1 to support tethering and rolling under natural conditions (Lawrence and Springer, 1991; von Andrian et al., 1991). These conditions do not include the cases where LFA-1 conformation was changed by mutations, I-domain isolation (Salas et al., 2002), or a small molecule XVA-143 (Salas et al., 2004). We suggest that these manipulations lead to a conformational change that facilitates LFA-1 ligand binding site exposure, and therefore, promotes rapid ligand-receptor engagement.

It worth noting that the possibility of a dramatic difference in the ligand kinetics between VLA-4 and LFA-1 (and Mac-1) was first suggested by Alon et al. (1995). These authors proposed that for VLA-4, which can mediate tethering and rolling without cell activation, rapid ligand association and dissociation rates would be observed. This conclusion was based on the analogy with other rolling receptors, i.e., selectins. These authors also suggested that for the LFA-1 interaction with ICAM-1, the ligand binding kinetics would be different (Alon et al., 1995). Now, experimental data directly supporting this concept are available (Chigaev et al., 2011b).

Because a major structural difference between VLA-4 and LFA-1 is the presence of an additional “inserted” I-domain, which was acquired by the I-domain containing leukocyte integrins at the time their emergence, it is tempting to attribute the difference in ligand binding kinetics to the presence of the domain. Without inside-out signaling, binding of ligand to LFA-1 is virtually absent, leading to the hypothesis that, at rest, the LFA-1 ligand binding site is “shielded” by some part of the molecule (Chigaev et al., 2011b). A rapid conformational rearrangement of LFA-1 upon activation (Shamri et al., 2005) could release this putative ligand binding site “protection,” and as a result mediate rapid receptor engagement. The presence of such intra- or intermolecular “protection” is supported by the fact that an isolated alpha L I-domain expressed on the cell surface was very effective in supporting cell rolling (Salas et al., 2002). We propose that a downward bending of the molecule that simply changes I-domain orientation would be insufficient to prevent binding of a small fluorescent ligand to LFA-1. In contrast, for VLA-4, the binding of the small fluorescent probe was not obstructed in its bent conformation. The VLA-4-specific small fluorescent ligand binding rate was close to its diffusion limit, where a FRET-based extension assay can be successfully performed (Chigaev et al., 2003a, 2004, 2007). We envision that a competitive protection mechanism that can be similar to an “endogenous ligand” (Alonso et al., 2002) could serve as a “shield” for the ICAM-1 binding site.

The physiological difference between the two integrins seems to be related to the function of the immune system. VLA-4 appears to be more important for innate antigen-independent immune responses, and LFA-1 for adaptive immunity. The presence of an additional protective mechanism for the binding of a ligand to the LFA-1 binding site suggests that LFA-1/ICAM-1-mediated interactions will be more difficult to establish. This is not surprising to researchers who performed side-by-side comparative studies of the two integrins. However, from a biological perspective, this seems to provide an additional “check” for adaptive immune responses, where intercellular immune cell interaction can directly lead to unwanted, or excessive immune activation and result in cell and tissue damage. This notion is additionally supported by the idea that the appearance of the leukocyte-specific alpha I domain-containing integrins (such as LFA-1) during vertebrate evolution coincides with the emergence of the BCR-TCR-MHC-based adaptive immune system.

One apparent exception from this observation is the crucial role of LFA-1 in chemokine-dependent arrest and trafficking of neutrophils, which is traditionally envisioned as a part of innate immunity. However, without questioning the well-established role of neutrophils in the rapid destruction of infectious agents, we would like to point toward emerging roles of neutrophils in immune regulation. As recent reports suggest, neutrophils can capture antigen and migrate to lymph nodes. They can also produce a repertoire of cytokines, chemokines, and angiogenic factors, provide signals for maturation of APCs, participate in the immune cells crosstalk that includes B and T cells, regulate adaptive immunity, and participate in the resolution of inflammation [for review see (Chakravarti et al., 2007; Kumar and Sharma, 2010; Mantovani et al., 2011)]. Thus, neutrophils should not be only envisioned as innate “weapons of mass destruction,” but also as emerging regulators of immune responses. Will some of these functions require LFA-1 integrin for the mediation of immune cell-cell interactions? We think that it is possible.

## Integrin physiology and its implication for drug discovery

Another remarkable difference between LFA-1 and VLA-4 integrins is the type of integrin antagonists identified in the attempt to regulate integrin dependent adhesion for therapeutic purposes. A majority of compounds specific for VLA-4 and several other integrins, including αIIbβ3 and αvβ3, are competitive (direct) inhibitors (Shimaoka and Springer, 2003) (formally agonists that promote LIBS). Until recently, no VLA-4-specific allosteric antagonists had been described (Chigaev et al., 2011d). For a competitive drug, the ligand binding site location is very close (or overlaps) with its natural ligand binding site. Therefore, direct competition with the integrin natural ligand can be observed. On the other hand, a large number of LFA-1 specific compounds are allosteric antagonists for two different allosteric sites on LFA-1 (Shimaoka and Springer, 2003). Is there a plausible explanation that can account for such distinction? Can different ligand binding properties provide an insight into such a peculiar difference?

Direct competitive inhibitors are expected to be ineffective in blocking LFA-1-dependent cell adhesion, if on resting cells, the LFA-1 binding domain is “hidden” and only exposed after inside-out activation. The binding of these compounds to LFA-1 would only be possible after an inside-out signal. Because LFA-1 activation and engagement can occur locally, right on the spot, where activating receptors, LFA-1, and ICAM-1 are juxtaposed at the site of contact (Shamri et al., 2005; Laudanna and Alon, 2006), competitive inhibitors would be highly inefficient in competition with natural ligands. For integrins possessing a ligand binding site that is exposed at rest (such as VLA-4), binding of competitive inhibitors would occur at any time, and binding site occupancy would simply depend on binding affinity and drug concentration.

On the other hand, binding of allosteric antagonists to their binding site, which is spatially separated from the ligand binding pocket, should be independent of the natural binding site exposure. Therefore, these compounds can occupy LFA-1 prior to its activation, and thus, should be more efficient in blocking LFA-1-dependent cell adhesion. We postulate that because of this property, in screening assays aimed at identifying LFA-1-specific antagonists, the number of allosteric “hits” was artificially enriched. This resulted in the predominance of LFA-1-specific allosteric antagonists (Shimaoka and Springer, 2003).

Is it possible to identify allosteric antagonists for integrins with an exposed ligand binding site? Using an approach that relies upon the exposure of the Ligand Induced Binding Site epitope (LIBS) to distinguish VLA-4 competitive antagonists (Njus et al., 2009), several VLA-4-specific allosteric antagonists were identified (Chigaev et al., 2011d). These molecules, although not competing directly with VLA-4-specific ligands, blocked VLA-4-dependent cell adhesion. Moreover, they mobilized early hematological progenitors into the peripheral blood, which is a well-documented ability of anti-VLA-4 blocking antibodies or competitive inhibitors (Bonig et al., 2008, 2009; Zohren et al., 2008; Ramirez et al., 2009). Moreover, because several of the identified molecules are FDA approved drugs that have been used over the past 30 years for treatment of non-hematological diseases, it appears that these anti-VLA-4 allosteric properties account for the previously reported hematological side effects (Chigaev et al., 2011c).

## Conclusions

An evolutionary divergence among ancient and more modern leukocyte integrins, containing an inserted alpha I-domain, provides a plausible mechanism to account for structural and functional differences between VLA-4 and LFA-1. A new set of fluorescent approaches has made it possible to study the affinity and conformation of these integrins in real-time on live cells at natural receptor abundance using several homogeneous assays. The ability of VLA-4 to bind ligand in the low affinity resting state as well as the high affinity activated state allows it to serve as an adhesion receptor for rolling as well as firm attachment. The inability of LFA-1 to bind ligand in its natural resting state suggests that its normal function is as a firm attachment receptor in conjunction with selectins as rolling receptors. These ligand binding differences provide an explanation to account for the fact that VLA-4 inhibitors are typically competitive, while inhibitors for LFA-1 are typically allosteric. Moreover, the ability of integrins to independently regulate molecular extension as well as affinity through known physiological pathways suggests a means for independent regulation of the adhesive capture efficiency as compared to adhesive duration.

### Conflict of interest statement

The authors declare that the research was conducted in the absence of any commercial or financial relationships that could be construed as a potential conflict of interest.

## References

[B1] Alon R. Kassner P. D. Carr M. W. Finger E. B. Hemler M. E. Springer T. A. (1995). The integrin VLA-4 supports tethering and rolling in flow on VCAM-1. J. Cell Biol. 128, 1243–1253 7534768 10.1083/jcb.128.6.1243 PMC2120426

[B2] Alonso J. L. Essafi M. Xiong J. P. Stehle T. Arnaout M. A. (2002). Does the integrin alphaA domain act as a ligand for its betaA domain? Curr. Biol. 12, R340–R342 10.1016/S0960-9822(02)00852-7 12015130

[B3] Askari J. A. Buckley P. A. Mould A. P. Humphries M. J. (2009). Linking integrin conformation to function. J. Cell Sci. 122, 165–170 10.1242/jcs.018556 19118208 PMC2714414

[B4] Berlin C. Bargatze R. F. Campbell J. J. von Andrian U. H. Szabo M. C. Hasslen S. R. Nelson R. D. Berg E. L. Erlandsen S. L. Butcher E. C. (1995). alpha 4 integrins mediate lymphocyte attachment and rolling under physiologic flow. Cell 80, 413–422 10.1016/0092-8674(95)90491-3 7532110

[B5] Bonig H. Watts K. L. Chang K. H. Kiem H. P. Papayannopoulou T. (2009). Concurrent blockade of alpha4-integrin and CXCR4 in hematopoietic stem/progenitor cell mobilization. Stem Cells 27, 836–837 10.1002/stem.9 19350684 PMC2892056

[B6] Bonig H. Wundes A. Chang K. H. Lucas S. Papayannopoulou T. (2008). Increased numbers of circulating hematopoietic stem/progenitor cells are chronically maintained in patients treated with the CD49d blocking antibody natalizumab. Blood 111, 3439–3441 10.1182/blood-2007-09-112052 18195093 PMC2275012

[B7] Bos J. L. (2006). Epac proteins: multi-purpose cAMP targets. Trends Biochem. Sci. 31, 680–686 10.1017/S0031182011001685 17084085

[B8] Burkhardt J. K. (2008). Integrins put the brakes on microcluster dynamics at the immunological synapse. Immunity 28, 732–734 10.1016/j.immuni.2008.05.002 18549795

[B9] Carrasco Y. R. Batista F. D. (2006). B-cell activation by membrane-bound antigens is facilitated by the interaction of VLA-4 with VCAM-1. EMBO J. 25, 889–899 10.1038/sj.emboj.7600944 16456548 PMC1383545

[B10] Chakravarti A. Allaeys I. Poubelle P. E. (2007). Neutrophils and immunity: is it innate or acquired? Med. Sci. (Paris) 23, 862–867 10.1051/medsci/20072310862 17937896

[B11] Chigaev A. Blenc A. M. Braaten J. V. Kumaraswamy N. Kepley C. L. Andrews R. P. Oliver J. M. Edwards B. S. Prossnitz E. R. Larson R. S. Sklar L. A. (2001). Real time analysis of the affinity regulation of alpha 4-integrin. The physiologically activated receptor is intermediate in affinity between resting and Mn(2+) or antibody activation. J. Biol. Chem. 276, 48670–48678 10.1074/jbc.M103194200 11641394

[B12] Chigaev A. Buranda T. Dwyer D. C. Prossnitz E. R. Sklar L. A. (2003a). FRET detection of cellular alpha4-integrin conformational activation. Biophys. J. 85, 3951–3962 10.1016/S0006-3495(03)74809-7 14645084 PMC1303696

[B13] Chigaev A. Zwartz G. Graves S. W. Dwyer D. C. Tsuji H. Foutz T. D. Edwards B. S. Prossnitz E. R. Larson R. S. Sklar L. A. (2003b). Alpha4beta1 integrin affinity changes govern cell adhesion. J. Biol. Chem. 278, 38174–38182 10.1074/jbc.M210472200 12844491

[B14] Chigaev A. Sklar L. A. (2012). Overview: assays for studying integrin-dependent cell adhesion. Methods Mol. Biol. 757, 3–14 10.1007/978-1-61779-166-6_1 21909902 PMC3805125

[B15] Chigaev A. Smagley Y. Sklar L. A. (2011a). Nitric oxide/cGMP pathway signaling actively down-regulates alpha4beta1-integrin affinity: an unexpected mechanism for inducing cell de-adhesion. BMC Immunol. 12, 28 10.1186/1471-2172-12-28 21586157 PMC3125286

[B16] Chigaev A. Smagley Y. Zhang Y. Waller A. Haynes M. K. Amit O. Wang W. Larson R. S. Sklar L. A. (2011b). Real-time analysis of the inside-out regulation of lymphocyte function-associated antigen-1 revealed similarities to and differences from very late antigen-4. J. Biol. Chem. 286, 20375–20386 10.1074/jbc.M110.206185 21515675 PMC3121518

[B17] Chigaev A. Winter S. S. Sklar L. A. (2011c). Is prolonged stem cell mobilization detrimental for hematopoiesis? Med. Hypotheses 77, 1111–1113 10.1016/j.mehy.2011.09.015 21963354 PMC3210378

[B18] Chigaev A. Wu Y. Williams D. B. Smagley Y. Sklar L. A. (2011d). Discovery of very late antigen-4 (VLA-4, α4β1 integrin) allosteric antagonists. J. Biol. Chem. 286, 5455–5463 10.1074/jbc.M110.162636 21131351 PMC3037658

[B19] Chigaev A. Waller A. Amit O. Halip L. Bologa C. G. Sklar L. A. (2009). Real-time analysis of conformation-sensitive antibody binding provides new insights into integrin conformational regulation. J. Biol. Chem. 284, 14337–14346 10.1074/jbc.M901178200 19251697 PMC2682882

[B20] Chigaev A. Waller A. Amit O. Sklar L. A. (2008). Galphas-coupled receptor signaling actively down-regulates alpha4beta1-integrin affinity: a possible mechanism for cell de-adhesion. BMC Immunol. 9, 26. 10.1186/1471-2172-9-26 18534032 PMC2442041

[B21] Chigaev A. Waller A. Zwartz G. J. Buranda T. Sklar L. A. (2007). Regulation of cell adhesion by affinity and conformational unbending of alpha4beta1 integrin. J. Immunol. 178, 6828–6839 17513731 10.4049/jimmunol.178.11.6828

[B22] Chigaev A. Zwartz G. J. Buranda T. Edwards B. S. Prossnitz E. R. Sklar L. A. (2004). Conformational regulation of alpha 4 beta 1-integrin affinity by reducing agents. “Inside-out” signaling is independent of and additive to reduction-regulated integrin activation. J. Biol. Chem. 279, 32435–32443 10.1074/jbc.M404387200 15166232

[B23] Coulombel L. Auffray I. Gaugler M. H. Rosemblatt M. (1997). Expression and function of integrins on hematopoietic progenitor cells. Acta Haematol. 97, 13–21 8980606 10.1159/000203655

[B24] DiVietro J. A. Brown D. C. Sklar L. A. Larson R. S. Lawrence M. B. (2007). Immobilized stromal cell-derived factor-1alpha triggers rapid VLA-4 affinity increases to stabilize lymphocyte tethers on VCAM-1 and subsequently initiate firm adhesion. J. Immunol. 178, 3903–3911 17339490 10.4049/jimmunol.178.6.3903

[B25] Dustin M. L. (2008). Hunter to gatherer and back: immunological synapses and kinapses as variations on the theme of amoeboid locomotion. Annu. Rev. Cell Dev. Biol. 24, 577–596 10.1146/annurev.cellbio.24.110707.175226 18598213

[B26] Flajnik M. F. Kasahara M. (2010). Origin and evolution of the adaptive immune system: genetic events and selective pressures. Nat. Rev. Genet. 11, 47–59 10.1038/nrg2703 19997068 PMC3805090

[B27] Franitza S. Grabovsky V. Wald O. Weiss I. Beider K. Dagan M. Darash-Yahana M. Nagler A. Brocke S. Galun E. Alon R. Peled A. (2004). Differential usage of VLA-4 and CXCR4 by CD3+CD56+ NKT cells and CD56+CD16+ NK cells regulates their interaction with endothelial cells. Eur. J. Immunol. 34, 1333–1341 10.1002/eji.200324718 15114666

[B28] Gadek T. R. Burdick D. J. McDowell R. S. Stanley M. S. Marsters J. C. Jr. Paris K. J. Oare D. A. Reynolds M. E. Ladner C. Zioncheck K. A. Lee W. P. Gribling P. Dennis M. S. Skelton N. J. Tumas D. B. Clark K. R. Keating S. M. Beresini M. H. Tilley J. W. Presta L. G. Bodary S. C. (2002). Generation of an LFA-1 antagonist by the transfer of the ICAM-1 immunoregulatory epitope to a small molecule. Science 295, 1086–1089 10.1126/science.295.5557.1086 11834839

[B29] Gazitt Y. (2004). Homing and mobilization of hematopoietic stem cells and hematopoietic cancer cells are mirror image processes, utilizing similar signaling pathways and occurring concurrently: circulating cancer cells constitute an ideal target for concurrent treatment with chemotherapy and antilineage-specific antibodies. Leukemia 18, 1–10 10.1038/sj.leu.2403173 14574330

[B30] Grabovsky V. Feigelson S. Chen C. Bleijs D. A. Peled A. Cinamon G. Baleux F. Arenzana-Seisdedos F. Lapidot T. van Kooyk Y. Lobb R. R. Alon R. (2000). Subsecond induction of alpha4 integrin clustering by immobilized chemokines stimulates leukocyte tethering and rolling on endothelial vascular cell adhesion molecule 1 under flow conditions. J. Exp. Med. 192, 495–506 10.1084/jem.192.4.495 10952719 PMC2193239

[B31] Hogg N. Patzak I. Willenbrock F. (2011). The insider's guide to leukocyte integrin signalling and function. Nat. Rev. Immunol. 11, 416–426 10.1038/nri2986 21597477

[B32] Huhtala M. Heino J. Casciari D. de Luise A. Johnson M. S. (2005). Integrin evolution: insights from ascidian and teleost fish genomes. Matrix Biol. 24, 83–95 10.1016/j.matbio.2005.01.003 15890260

[B33] Imai Y. Shimaoka M. Kurokawa M. (2010). Essential roles of VLA-4 in the hematopoietic system. Int. J. Hematol. 91, 569–575 10.1007/s12185-010-0555-3 20352381

[B34] Kadioglu A. De Filippo K. Bangert M. Fernandes V. E. Richards L. Jones K. Andrew P. W. Hogg N. (2011). The integrins Mac-1 and alpha4beta1 perform crucial roles in neutrophil and T cell recruitment to lungs during *Streptococcus pneumoniae* infection. J. Immunol. 186, 5907–5915 10.4049/jimmunol.1001533 21460207

[B35] Kong F. Garcia A. J. Mould A. P. Humphries M. J. Zhu C. (2009). Demonstration of catch bonds between an integrin and its ligand. J. Cell Biol. 185, 1275–1284 10.1083/jcb.200810002 19564406 PMC2712956

[B36] Kumar V. Sharma A. (2010). Neutrophils: cinderella of innate immune system. Int. Immunopharmacol. 10, 1325–1334 10.1016/j.intimp.2010.08.012 20828640

[B37] Kuwano Y. Spelten O. Zhang H. Ley K. Zarbock A. (2010). Rolling on E- or P-selectin induces the extended but not high-affinity conformation of LFA-1 in neutrophils. Blood 116, 617–624 10.1182/blood-2010-01-266122 20445017 PMC3324292

[B38] Larson R. S. Davis T. Bologa C. Semenuk G. Vijayan S. Li Y. Oprea T. Chigaev A. Buranda T. Wagner C. R. Sklar L. A. (2005). Dissociation of I domain and global conformational changes in LFA-1, refinement of small molecule-I domain structure-activity relationships. Biochemistry 44, 4322–4331 10.1021/bi048187k 15766261

[B39] Laudanna C. Alon R. (2006). Right on the spot. Chemokine triggering of integrin-mediated arrest of rolling leukocytes. Thromb. Haemost. 95, 5–11 10.1160/TH05-07-0482 16543955

[B40] Lawrence M. B. Springer T. A. (1991). Leukocytes roll on a selectin at physiologic flow rates: distinction from and prerequisite for adhesion through integrins. Cell 65, 859–873 10.1016/0092-8674(91)90393-D 1710173

[B41] Lefort C. T. Rossaint J. Moser M. Petrich B. G. Zarbock A. Monkley S. J. Critchley D. R. Ginsberg M. H. Fassler R. Ley K. (2012). Distinct roles for talin-1 and kindlin-3 in LFA-1 extension and affinity regulation. Blood 119, 4275–4282 10.1182/blood-2011-08-373118 22431571 PMC3359742

[B42] Lopez-Hoyos M. Revilladagger C. Conde C. Del Campo E. G. Gonzalez A. Merino J. (1999). Different roles for LFA-1 and VLA-4 integrins in T-B-cell interactions *in vivo*. Immunology 97, 438–446 10447765 10.1046/j.1365-2567.1999.00794.x PMC2326849

[B43] Mantovani A. Cassatella M. A. Costantini C. Jaillon S. (2011). Neutrophils in the activation and regulation of innate and adaptive immunity. Nat. Rev. Immunol. 11, 519–531 10.1038/nri3024 21785456

[B44] Mould A. P. Barton S. J. Askari J. A. McEwan P. A. Buckley P. A. Craig S. E. Humphries M. J. (2003). Conformational changes in the integrin beta A domain provide a mechanism for signal transduction via hybrid domain movement. J. Biol. Chem. 278, 17028–17035 10.1074/jbc.M213139200 12615914

[B45] Mould A. P. Humphries M. J. (2004). Regulation of integrin function through conformational complexity: not simply a knee-jerk reaction? Curr. Opin. Cell Biol. 16, 544–551 10.1016/j.ceb.2004.07.003 15363805

[B46] Njus B. H. Chigaev A. Waller A. Wlodek D. Ostopovici-Halip L. Ursu O. Wang W. Oprea T. I. Bologa C. G. Sklar L. A. (2009). Conformational mAb as a tool for integrin ligand discovery. Assay Drug Dev. Technol. 7, 507–515 10.1089/adt.2009.0203 19754304 PMC3096548

[B47] Oostendorp R. A. Dormer P. (1997). VLA-4-mediated interactions between normal human hematopoietic progenitors and stromal cells. Leuk. Lymphoma 24, 423–435 10.3109/10428199709055581 9086434

[B48] Peled A. Kollet O. Ponomaryov T. Petit I. Franitza S. Grabovsky V. Slav M. M. Nagler A. Lider O. Alon R. Zipori D. Lapidot T. (2000). The chemokine SDF-1 activates the integrins LFA-1, VLA-4, and VLA-5 on immature human CD34(+) cells: role in transendothelial/stromal migration and engraftment of NOD/SCID mice. Blood 95, 3289–3296 10828007

[B49] Prossnitz E. R. (1997). Desensitization of N-formylpeptide receptor-mediated activation is dependent upon receptor phosphorylation. J. Biol. Chem. 272, 15213–15219 10.1074/jbc.272.24.15213 9182544

[B50] Ramirez P. Rettig M. P. Uy G. L. Deych E. Holt M. S. Ritchey J. K. DiPersio J. F. (2009). BIO5192, a small molecule inhibitor of VLA-4, mobilizes hematopoietic stem and progenitor cells. Blood 114, 1340–1343 10.1182/blood-2008-10-184721 19571319 PMC2727418

[B51] Salas A. Shimaoka M. Chen S. Carman C. V. Springer T. (2002). Transition from rolling to firm adhesion is regulated by the conformation of the I domain of the integrin lymphocyte function-associated antigen-1. J. Biol. Chem. 277, 50255–50262 10.1074/jbc.M209822200 12368274

[B52] Salas A. Shimaoka M. Kogan A. N. Harwood C. von Andrian U. H. Springer T. A. (2004). Rolling adhesion through an extended conformation of integrin alphaLbeta2 and relation to alpha I and beta I-like domain interaction. Immunity 20, 393–406 10.1016/S1074-7613(04)00082-2 15084269

[B53] Shamri R. Grabovsky V. Gauguet J. M. Feigelson S. Manevich E. Kolanus W. Robinson M. K. Staunton D. E. von Andrian U. H. Alon R. (2005). Lymphocyte arrest requires instantaneous induction of an extended LFA-1 conformation mediated by endothelium-bound chemokines. Nat. Immunol. 6, 497–506 10.1038/ni1194 15834409

[B54] Shimaoka M. Springer T. A. (2003). Therapeutic antagonists and conformational regulation of integrin function. Nat. Rev. Drug Discov. 2, 703–716 10.1038/nrd1174 12951577

[B55] Springer T. A. (1994). Traffic signals for lymphocyte recirculation and leukocyte emigration: the multistep paradigm. Cell 76, 301–314 10.1016/0092-8674(94)90337-9 7507411

[B56] Springer T. A. Dustin M. L. (2012). Integrin inside-out signaling and the immunological synapse. Curr. Opin. Cell Biol. 24, 107–115 10.1016/j.ceb.2011.10.004 22129583 PMC3294052

[B57] von Andrian U. H. Chambers J. D. McEvoy L. M. Bargatze R. F. Arfors K. E. Butcher E. C. (1991). Two-step model of leukocyte-endothelial cell interaction in inflammation: distinct roles for LECAM-1 and the leukocyte beta 2 integrins *in vivo*. Proc. Natl. Acad. Sci. U.S.A. 88, 7538–7542 1715568 10.1073/pnas.88.17.7538 PMC52336

[B58] Wang Q. Q. Li H. Oliver T. Glogauer M. Guo J. He Y. W. (2008). Integrin beta 1 regulates phagosome maturation in macrophages through Rac expression. J. Immunol. 180, 2419–2428 18250451 10.4049/jimmunol.180.4.2419

[B59] Warnock R. A. Askari S. Butcher E. C. von Andrian U. H. (1998). Molecular mechanisms of lymphocyte homing to peripheral lymph nodes. J. Exp. Med. 187, 205–216 10.1084/jem.187.2.205 9432978 PMC2212097

[B60] Zohren F. Toutzaris D. Klarner V. Hartung H. P. Kieseier B. Haas R. (2008). The monoclonal anti-VLA-4 antibody natalizumab mobilizes CD34+ hematopoietic progenitor cells in humans. Blood 111, 3893–3895 10.1182/blood-2007-10-120329 18235044

[B61] Zwartz G. Chigaev A. Foutz T. Larson R. S. Posner R. Sklar L. A. (2004). Relationship between molecular and cellular dissociation rates for VLA-4/VCAM-1 interaction in the absence of shear stress. Biophys. J. 86, 1243–1252 10.1016/S0006-3495(04)74198-3 14747358 PMC1303916

